# Supplementary material for: Impact of Almond Variety on “Amaretti” Cookies as Assessed through Image Features Modeling, Physical Chemical Measures and Sensory Analyses
Source: Foods. 2020 Sep 10;9(9):1272. doi: 10.3390/foods9091272 (PMC7556034; doi:10.3390/foods9091272)
Supplement: Supplementary file 1 [file foods-09-01272-s001.pdf]

CONTENTS

Supplementary Tables 3

1. **Table S1: Sensory attributes, definition and reference standards used for Amaretti cookies** 3
2. **Table S2: Mean and standard deviation of sensory descriptors of Amaretti valued at 1 day of storage.** 4
3. **Table S3: Mean and standard deviation of sensory descriptors of *Amaretti* valued at 7 days of storage.** 5
4. **Table S4: Mean and standard deviation of sensory descriptors of Amaretti valued at 30 days of storage.** 6
5. **Table S5: Mean and standard deviation of sensory descriptors of Amaretti valued at 60 days of storage.** 7
6. **Table S6: Sensory acceptance of sample after 0, 7, 30, and 60 days of storage.**

Table S1. Sensory attributes, definition and reference standards used for "Amaretti" cookies

Attributes		Definition	Reference Standards (and their position on the scale)
**Appearance**	Color	Color hue from light beige to dark beige	Photo
	Visual roughness	Degree of roughness visible on the surface of the sample	Apple Granny Smith-Savoiardi biscuits-Barilla Granetti (2, 5 and 9)
	Tactile hardness	Force required to deformation or penetration of a product using the fingers	Savoiardi biscuits-Barilla Granetti (2 and 9)
**Odor**	Amaretto Benzaldehyde	Odors compounds associated with benzaldehyde; reminiscent of cherry or some almonds	PANEANGELI (almond aroma) 1 drop/50 mL water, 5 drops/30 mL water (3 and 8)
	Citrus fruits	Odors compounds associated with the citrus category	PANEANGELI (citrus fruit aroma) 1 drop/30 mL water, 5 drops/30 mL water (3 and 7)
	Sweet	Odors associated with that smell sweet, such as honey, brown sugar and vanilla.	Mixture of brown sugar, honey, vanilla, flour, butter and water. 20, 10, 0,1, 20, 20 g and 50 mL, respectively (9)

	Caramel	Odors compounds associated with product that smell of caramelized sugar	FABBRI (caramel aroma) Fabbri caramel 5 mL/50 mL water, FABBRI caramel (1 and 8)
**Texture**	Hardness	Force required to achieved a given deformation or penetration of a product using the molars	Barilla Plumcake-Barilla Tarallucci (1 and 8)
	Friability	Amaunt of particles formed after 4/5 chewing	Barilla Plumcake-Barilla Tarallucci (1 and 8)
	Moistness	Perception of moisture content of a food by the tactile recptors in the mouth	Meringue, Barilla Plumcake, Apple Granny Smith (1, 5 and 8)
	Adhesiveness	Force required to remove material that sticks to the mouth and teeth	Hard-boiled egg white, Barilla Plumcake, Mars (1, 4 and 8)
	Solubility	Perception when the product melts rapidly in saliva	Hard-boiled egg white, Barilla Plumcake, Meringue (1, 5 and 9)
**Aroma**	Amaretto Benzaldehyde	Aromatics associated with benzaldehyde; reminiscent of cherry or some almonds	PANEANGELI (almond aroma) 1 drop/30 mL water, 6 drops/30 mL water (3 and 9)
	Citrus fruits	Aromatics associated with the citrus category	PANEANGELI (citrus fruit aroma) 1 drop/30 mL water, 6 drops/30 mL water (3 and 9)
	Bitter almond	Aromatics associated with the bitter almonds	Slightly bitter almond and bitter almond (2 and 8)
	Off-Flavour	Atypical flavor often associated with the deterioration or transformation of the product	
**Taste**	Sweetness	Primary taste produced by aqueous solutions of substances such as sucrose	10 and 100 g sucrose/L (2 and 8)
	Bitterness	Primary taste produced by aqueous solutions of substances such as caffeine	0.3 and 0.6 g caffeine/L (2 and 8)

Table S2. Mean and standard deviation of sensory descriptors of *Amaretti* valued at 1 day of storage.

**Descriptors**	**Alamonds Varieties**				**Significance**
	**AR T1**	**CO T1**	**TX T1**	**TU T1**	
Color	5.69±1.28	5.47±1.04	5.43±1.15	5.391.28	ns

Visual Roughness	6.43±0.75	6.16±0.97	6.34±1.00	6.49±0.76	ns
Tactile Hardness	[a]4.99±1.16	[a]5.35±1.10	[b]3.88±0.96	[a]5.31±1.38	$P < 0.018$
Amaretto odor	5.09±1.09	5.17±1.38	5.38±1.14	5.50±0.90	ns
Citrus fruits odor	3.96±1.14	4.47±1.26	4.52±1.42	4.59±0.94	ns
Sweet odor	5.27±1.47	5.86±1.20	5.54±1.46	5.41±1.56	ns
Caramel odor	1.38±0.99	1.72±1.03	1.58±1.02	1.69±1.04	ns
Hardness	[ab]3.06±1.41	[a]4.07±1.47	[b]2.81±1.07	[ab]3.19±1.19	$P < 0.043$
Friability	3.29±1.33	3.11±1.33	3.17±0.92	2.84±1.13	ns
Moistness	4.76±0.79	4.55±0.64	4.92±0.83	4.42±0.72	ns
Adhesiveness	[ab]4.86±0.74	[a]5.44±0.99	[ab]5.03±0.84	[b]4.45±1.22	$P < 0.046$
Solubility	4.84±0.85	4.26±0.71	4.79±0.99	4.52±0.90	ns
Amaretto aroma	6.35±1.20	5.98±1.36	6.36±1.31	5.83±1.12	ns
Citrus fruits aroma	5.32±2.06	5.61±1.89	5.80±1.71	5.50±1.74	ns
Bitter almond aroma	3.20±1.22	2.83±1.41	2.69±1.23	2.97±1.35	ns
Off-Flavour	0.82±1.79	0.79±1.20	0.63±1.38	0.56±0.83	ns
Sweet taste	6.02±0.83	5.41±1.85	5.64±1.86	5.64±0.88	ns
Bitter taste	2.78±1.58	2.31±1.33	2.24±1.36	2.07±0.85	ns

Different letters within the same line indicate significant differences among the sample (P <0.05). n.s.. not significant.

Table S3. Mean and standard deviation of sensory descriptors of *Amaretti* valued at 7 days of storage.

**Descriptors**	**Almonds Varieties**				**Significance**
	**AR T7**	**CO T7**	**TX T7**	**TU T7**	
Color	5.55±1.64	5.54±1.03	5.66±1.63	4.92±1.36	ns
Visual Roughness	6.22±0.76	6.12±0.76	6.25±0.77	6.18±0.91	ns
Tactile Hardness	6.25±1.37	6.97±0.97	6.45±1.29	6.61±1.22	ns
Amaretto odor	5.59±0.79	5.06±0.99	4.91±1.35	5.19±1.08	ns
Citrus fruits odor	4.39±1.01	4.05±1.09	4.02±0.99	4.25±1.20	ns
Sweet odor	5.07±1.62	5.34±1.78	5.08±1.85	5.42±1.64	ns
Caramel odor	1.62±1.19	1.55±1.27	1.44±1.11	1.75±1.31	ns
Hardness	3.38±1.35	4.43±0.80	3.55±1.38	3.82±1.29	ns
Friability	3.50±1.77	4.34±1.06	3.90±1.13	4.18±1.23	ns

Moistness	4.49±1.11	3.92±1.12	4.28±0.92	4.23±1.13	ns
Adhesiveness	4.33±1.24	4.46±1.43	4.37±1.17	4.39±1.49	ns
Solubility	4.92±0.99	4.50±0.97	4.93±0.93	4.86±0.82	ns
Amaretto aroma	6.08±1.93	6.13±1.29	5.58±1.25	6.32±1.22	ns
Citrus fruits aroma	5.28±1.41	4.74±1.58	5.14±1.17	5.30±1.43	ns
Bitter almond aroma	2.96±1.00	3.21±1.33	3.21±1.27	3.19±1.39	ns
Off-Flavour	0.74±1.47	0.79±1.03	0.74±1.26	0.65±0.99	ns
Sweet taste	6.23±0.48	5.78±0.98	5.89±1.05	6.11±0.67	ns
Bitter taste	2.30±1.26	2.77±1.64	2.46±1.49	2.42±1.35	ns

Different letters within the same line indicate significant differences among the sample (p <0.05). n.s.. not significant.

Table S4. Mean and standard deviation of sensory descriptors of *Amaretti* valued at 30 days of storage.

**Descriptors**	**Almonds Varieties**				**Significance**
	**AR T30**	**CO T30**	**TX T30**	**TU T30**	
Color	5.92±1.27	5.14±1.38	5.67±1.23	4.89±1.11	ns
Visual Roughness	6.74±1.30	6.01±1.36	6.77±1.10	6.00±2.12	ns
Tactile Hardness	^{ab}7.26±1.33	^{a}7.75±1.14	^{a}8.00±0.57	^{b}6.91±1.27	$P < 0.027$
Amaretto odor	^{ab}5.20±0.94	^{bc}4.68±1.06	^{c}4.32±1.35	^{a}5.50±0.99	$P < 0.012$
Citrus fruits odor	4.45±1.34	3.95±1.37	3.81±1.37	4.24±1.28	ns
Sweet odor	4.99±2.01	5.02±2.08	4.81±2.46	5.11±1.79	ns
Caramel odor	1.14±0.82	1.12±0.78	1.05±0.75	1.22±0.89	ns
Hardness	^{bc}4.95±1.33	^{ab}5.53±1.11	^{a}5.85±1.19	^{b}4.60±1.40	$P < 0.024$
Friability	5.08±1.33	5.21±1.02	5.48±1.02	4.75±1.24	ns
Moistness	3.92±1.14	3.57±1.02	3.39±1.05	4.12±1.00	ns
Adhesiveness	4.14±1.58	4.31±1.66	4.01±1.80	4.02±1.42	ns
Solubility	4.61±1.45	4.47±1.59	4.90±1.79	5.00±1.34	ns
Amaretto aroma	5.64±1.30	5.68±1.28	4.84±1.19	5.99±1.18	ns
Citrus fruits aroma	5.10±1.72	5.02±1.67	4.68±1.67	5.04±1.50	ns
Bitter almond aroma	2.96±0.97	3.20±1.16	2.69±1.35	2.91±1.06	ns
Off-Flavour	0.98±1.58	0.82±1.37	1.07±1.56	0.96±1.67	ns

Sweet taste	6.15±0.53	6.49±0.41	6.31±0.46	6.28±0.46	ns
Bitter taste	2.40±1.11	2.66±1.41	2.38±1.41	2.47±1.20	ns

Different letters within the same line indicate significant differences among the sample (p <0.05). n.s.. not significan

Table S5. Mean and standard deviation of sensory descriptors of *Amaretti* valued at 60 days of storage.

**Descriptors**	**Alamonds Varieties**				**Significance**
	**AR T60**	**CO T60**	**TX T60**	**TU T60**	
Color	5.92±1.23	5.45±1.55	4.72±1.53	5.23±1.29	ns
Visual Roughness	6.74±0.98	6.48±1.21	6.08±1.53	5.75±1.26	ns
Tactile Hardness	8.29±0.61	8.45±0.96	7.45±1.53	8.06±1.37	ns
Amaretto odor	4.49±1.09	4.42±1.40	4.59±0.84	4.84±1.67	ns
Citrus fruits odor	3.68±1.41	3.68±1.43	3.78±1.36	3.69±1.63	ns
Sweet odor	5.16±2.39	5.56±2.34	5.29±2.35	4.96±2.41	ns
Caramel odor	1.05±0.66	0.96±0.75	1.11±0.79	0.92±0.67	ns
Hardness	^{b}5.57±1.51	^{a}6.87±1.63	^{b}5.11±1.59	^{ab}5.72±2.20	$P < 0.024$
Friability	^{b}5.70±1.21	^{a}6.82±1.16	^{b}5.12±1.72	^{b}5.50±2.11	$P < 0.024$
Moistness	3.32±1.06	2.99±1.28	3.69±1.16	3.52±1.32	ns
Adhesiveness	4.42±1.82	4.23±1.93	4.28±1.46	4.33±1.73	ns
Solubility	5.11±1.68	5.16±1.96	5.00±1.64	4.91±1.72	ns
Amaretto aroma	5.60±1.37	5.34±1.69	5.46±1.20	5.58±1.66	ns
Citrus fruits aroma	4.66±1.91	4.54±1.77	4.98±1.79	4.45±1.98	ns
Bitter almond aroma	2.81±1.37	2.96±1.64	2.79±0.99	3.04±1.25	ns
Off-Flavour	1.78±1.98	2.00±2.11	1.85±2.13	2.28±2.71	ns
Sweet taste	6.16±1.03	6.26±0.63	6.29±0.86	6.26±0.83	ns
Bitter taste	2.66±1.07	2.73±1.49	2.30±0.99	2.72±1.27	ns

Different letters within the same line indicate significant differences among the sample (p <0.05). n.s.. not significant.

Table S6. Sensory acceptance of sample after 0. 7. 30. and 60 days of storage.

	**T0**				**T7**				**T30**				**T60**			
**Sample**	AR	CO	TX	TU	AR	CO	TX	TU	AR	CO	TX	TU	AR	CO	TX	TU
Appearance	7.57^{a}	7.61^{a}	7.66^{a}	7.59^{a}	7.72^{a}	7.57^{a}	7.67^{a}	7.74^{a}	7.38^{a}	7.24^{a}	7.24^{a}	7.55^{a}	6.02	6.29	6.29	6.14
Taste	7.45^{b}	6.91^{a}	7.49^{b}	7.22^{ab}	7.15^{a}	7.26^{a}	7.35^{a}	7.20^{a}	6.95^{a}	6.57^{a}	6.93^{a}	7.00^{a}	4.46^{ab}	5.17^{c}	4.89^{bc}	4.29^{a}
Flavor	7.51^{b}	7.00^{a}	7.34^{ab}	7.18^{ab}	7.07^{a}	7.26^{a}	7.11^{a}	7.31^{a}	6.59^{a}	6.53^{a}	6.90^{a}	6.93^{a}	6.59	6.53	6.9	6.93
Taste	7.14^{c}	6.11^{a}	6.95^{bc}	6.64^{b}	7.07^{a}	7.06^{a}	7.28^{a}	7.22^{a}	6.47^{b}	5.81^{a}	6.67^{b}	6.78^{b}	3.91^{a}	5.08^{b}	4.80^{b}	3.57^{a}
Overall acceptability	7.45^{b}	6.57^{a}	7.32^{b}	6.93^{a}	7.24^{a}	7.11^{a}	7.33^{a}	7.11^{a}	6.72^{ab}	6.29^{a}	6.78^{ab}	6.93^{b}	4.55^{a}	5.44^{b}	5.08^{b}	4.10^{a}

Mean Values. Different letters within the same line indicate significant differences among the sample ($p < 0.05$).
